# Nitrous Oxide Gas

**Published:** 1883-03

**Authors:** 


*Nitrous Oxide Gas.*—Dr. F. C. Reeve in the American Edition of *Holmes' Surgery* makes the following statement.—" Further experience has not changed the relative position or much enlarged the sphere of action of nitrous oxide. That it is the safest of all anæsthetics has been established beyond a question. In one institution where such administration is subject of record, this gas has been given over 100,000 times, and not only without a death, but without causing in a single instance symptoms sufficiently serious to necessitate transporting the patient home in a carriage. In the city of Philadelphia alone, it has been given over 133,000 times without a death and without any injurious result. Death cannot be justly attributed to it in more than four cases since its introduction.

